# Physiological and molecular changes of onion (*Allium cepa* L.) seeds under different aging conditions

**DOI:** 10.1186/s12870-024-04773-7

**Published:** 2024-02-03

**Authors:** Reza Kamaei, Mohammad Kafi, Reza Tavakkol Afshari, Saeid Malekzadeh Shafaroudi, Jafar Nabati

**Affiliations:** 1 Ferdowsi University of Mashhad, Mashhad, Iran; 2 Department of Agrotechnonogy, Ferdowsi University of Mashhad, Mashhad, Iran; 3 Department of Biotechnology and Plant Breeding, Ferdowsi University of Mashhad, Mashhad, Iran

**Keywords:** Electrolyte leakage, Gene expression, Germination percentage, Protein concentration, Total sugar

## Abstract

**Background:**

Onion seeds have limited storage capacity compared to other vegetable seeds. It is crucial to identify the mechanisms that induce tolerance to storage conditions and reduce seed deterioration. To address this goal, an experiment was conducted to evaluate changes in germination, biochemical, physiological, and molecular characteristics of onion seed landraces (Horand, Kazerun landraces and Zargan cultivar) at different aging levels (control, three-days and six-days accelerated aging, and natural aging for one year).

**Results:**

The findings suggest that there was an increase in glucose, fructose, total sugar, and electrolyte leakage in the Horand (HOR), Kazerun (KAZ) landraces, and Zarghan (ZAR) cultivar, with Kazerun exhibiting the greatest increase. The percentage and rate of germination of Kazerun decreased by 54% and 33%, respectively, in six-day accelerated aging compared to the control, while it decreased by 12% and 14%, respectively, in Horand. Protein content decreased with increasing levels of aging, with a decrease of 26% in Kazerun landrace at six days of aging, while it was 16% in Horand landrace. The antioxidant activities of catalase, superoxide dismutase, and glutathione peroxidase decreased more intensively in Kazerun. The expression of *AMY1*, *BMY1*, *CTR1*, and *NPR1* genes were lower in Kazerun landraces than in Horand and Zargan at different aging levels.

**Conclusions:**

The *AMY1*, *BMY1*, *CTR1*, and *NPR1* genes play a pivotal role in onion seed germination, and their downregulation under stressful conditions has been shown to decrease germination rates. In addition, the activity of CAT, SOD, and GPx enzymes decreased by seed aging, and the amount of glucose, fructose, total sugar and electrolyte leakage increased, which ultimately led to seed deterioration. Based on the results of this experiment, it is recommended to conduct further studies into the molecular aspects involved in onion seed deterioration. More research on the genes related to this process is suggested, as well as investigating the impact of different priming treatments on the genes expression involved in the onion seed aging process.

## Background

Onion (*Allium cepa* L.) is a biennial crop from the Amaryllidaceae family and is one of the oldest cultivated vegetables in the world [1]. Onion seeds have limited storage capacity and higher moisture content than other conventional vegetables, making them susceptible to deterioration under inappropriate storage conditions [2]. This can reduce the viability, germination, and quality of the seeds, which makes it crucial to identify cultivars or landraces that are resistant to seed deterioration [3].

Seed deterioration is caused by various factors, including genetic factors, mechanical damage, relative humidity, storage temperature, seed moisture content, presence of micro flora, seed ripening and other factors that render the conditions unsuitable for seed quality retention [4]. Seed deterioration involves biochemical and physiological changes, including genetic modification, enzyme activity changes, and membrane damage, leading to a decrease in the germination rate, abnormal growth, and growth of seedlings [2].

The mechanisms involved in the aging process include changes in seed proteins, reactive oxygen species (ROS), changes in lipids, and in DNA [5]. Partial reduction of molecular oxygen creates ROS, such as superoxide radical, hydrogen peroxide, and hydroxyl radical [6], which have a destructive role in seeds due to their high affinity for biological molecules, including proteins, soluble sugars, lipids, and nucleic acids [7].

Seed aging has been reported to decrease the activity of peroxidase, catalase, ascorbate peroxidase, superoxide dismutase, and lipoxygenase enzymes as antioxidant systems [8]. In a study on rapeseed, Khan et al. [9] reported a significant decrease in the amount of soluble protein with the increase of seed decay. They also reported a decrease in the activity of antioxidant enzymes with the increase of seed decay. Brar et al. [10] reported that the activities of catalase, peroxidase, and superoxide dismutase enzymes decreased significantly with the increase in seed deterioration. On the other hand, the presence of sugars such as fructose, galactose, and glucose serve as the primary driving force for Amadori and Maillard reactions [11]. In seeds, sugars are formed during the hydrolysis of raffinose and stachyose during storage and are rapidly employed in Amadori and Maillard reactions [12]. It has been reported that the accumulation of Maillard products correlates strongly with glucose levels and structural deterioration of seeds, indicating that the hydrolysis of sugars during storage may be involved in seed deterioration due to preparatory and Maillard reactions [13].

Seed decay causes excessive or decreased expression of certain genes. Identifying the genes involved in modulating the effects of deterioration provides a way to identify the mechanisms involved in reducing the effects of deterioration in sensitive seeds. Until now, no study has been conducted on changes in the expression of genes involved in onion seed germination under the influence of seed decay. However, some studies have evaluated the expression of genes in other seeds such as in rapeseed (*AMYI*) [9], soybean (*AMYI* and *BMYI*) [14 and 18], Arabidopsis (*CTR1*) [15], and Arabidopsis (*NPR1*) under seed aging [16]. The researchers reported an increase in gene expression levels with an increase in seed degradation [14].

Onion seed storage is at risk due to the negative effects of temperature and humidity on its germination during long-term storage. Therefore, it is imperative to identify the mechanisms that promote resistance to seed deterioration. The objectives of this study are therefore: (1) to explore the mechanisms that contribute to tolerance against onion seed decay, (2) to compare the susceptible and resistant onion seeds in relation to physiological and molecular mechanisms, and (3) to identify potential indicators to control onion seed decay.

## Results

### Germination percentage

**Fig. 1:**
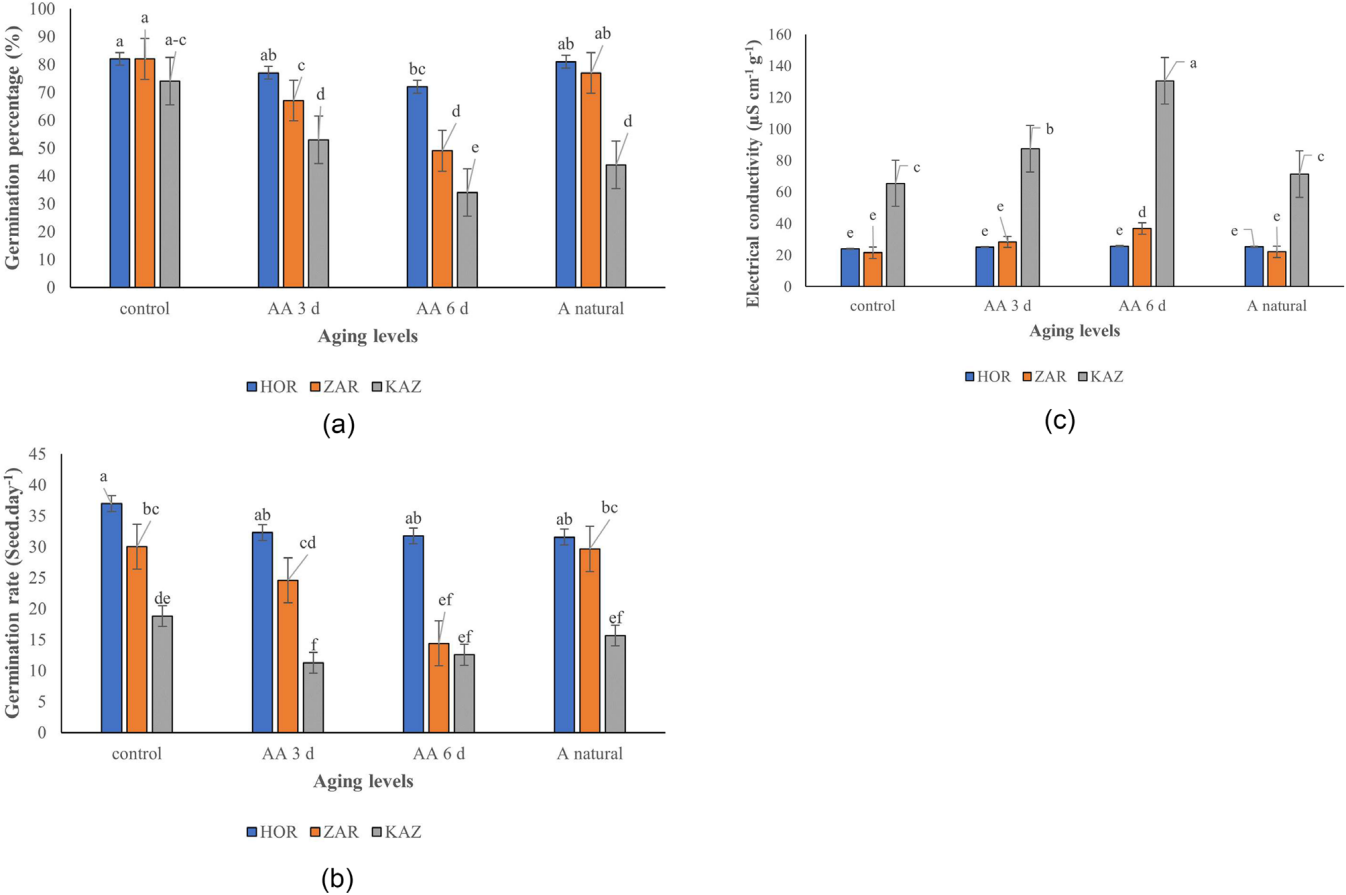
Germination percentage (**a**), Germination rate (**b**) and Electrical conductivity (**c**) of HOR, ZAR and KAZ onion landraces under different levels of aging [Means with the same letters are not significantly different using Duncan multiple range test (*p* value ≤ 0.05)]. AA 3d, AA6 d and A natural refer to three-days aging, six-days aging, and one-year natural aging, respectively

The results showed that the seed germination percentage was significantly affected by aging, landraces, and their interaction (*p* ≤ 0.001). Germination percentage decreased as the aging period increased, with a sharp decline observed in seeds subjected to 6-day accelerated aging (Fig. 1a). For Kazerun, the germination percentage decreased by 28%, 54%, and 41% in seeds subjected to 3-day aging, 6-day aging, and 1-year natural aging, compared to the control treatment, respectively. The germination percentage of Horand decreased by 6%, 12%, and 1% in 3-day aging, 6-day aging, and 1-year natural aging, compared to the control treatment, respectively. While for Zargan cultivar, the germination percentage decreased by 18%, 40%, and 6% in 3-day aging, 6-day aging, and 1-year natural aging, compared to the control treatment, respectively.

### Germination rate

The germination rate was also significantly affected by aging, landraces, and their interaction (*p* ≤ 0.001). The germination rate decreased as the aging period increased (Fig. 1b). The germination rate of Horand decreased by 13%, 14%, and 15% at 3-day aging, 6-day aging, and 1-year natural aging, respectively, while that of Zargan cultivar decreased by 18%, 52%, and 1%. Kazerun showed a decrease of 40%, 33%, and 17% in seeds germination subjected to 3-day aging, 6-day aging, and 1-year natural aging, compared to the control, respectively.

### Electrolyte leakage

**Fig. 2:**
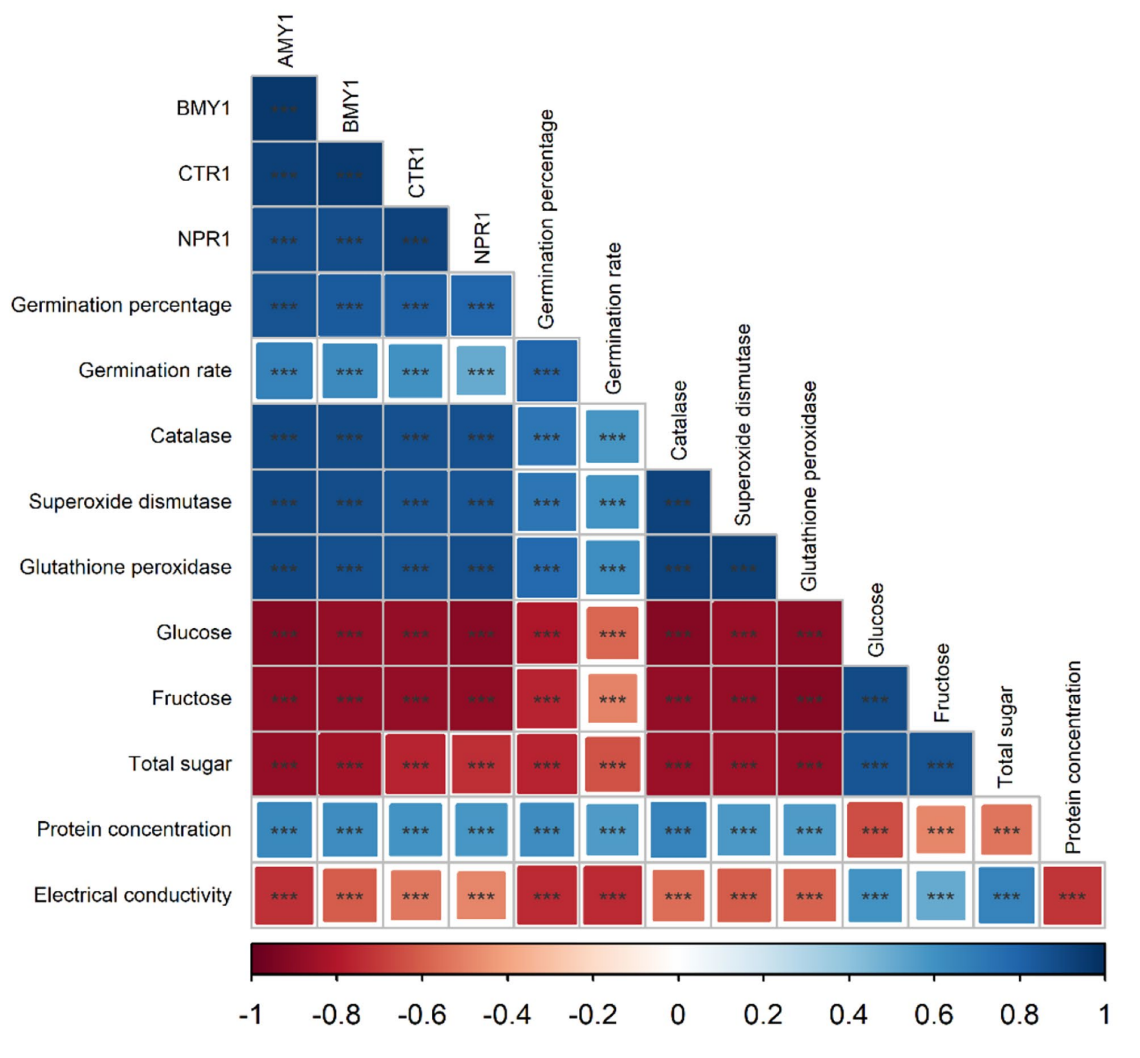
Pearson correlation matrix of germination percentage, germination rate, catalase, superoxide dismutase, glutathione peroxidase, glucose, fructose, total sugar, protein concentration, electrical conductivity, *AMY1*, *BMY1*, *CTR1* and *NPR1* gene expressions. (*, **, and ***; significant difference at *p* ≤ 0.05, *p* ≤ 0.01, and *p* ≤ 0.001, respectively)

The data on electrolyte leakage was analyzed using ANOVA and showed significant effects of seed aging, landraces, and their interaction (*p* ≤ 0.001). Electrolyte leakage increased at all levels of aging, with the highest increase observed in the Kazerun landrace. Specifically, in Kazerun, electrolyte leakage increased by 33%, 99%, and 9% at three-day aging, six-day aging, and one-year natural aging, compared to the control, respectively. Similarly, in Zargan cultivar, electrolyte leakage increased by 31%, 71%, and 2% in three-day aging, six-day aging, and one-year natural aging, compared to the control, respectively. However, there was no significant difference between different aging levels in Horand in terms of electrolyte leakage. These results suggest that electrolyte leakage is affected by both aging and landraces, while the Kazerun being the most sensitive to aging-induced changes in electrolyte leakage (Fig. 1c). In this study, we observed a negative correlation between electrolyte leakage, germination percentage, and germination rate (Fig. 2).

### The seed soluble sugars contents

#### Glucose

**Fig. 3:**
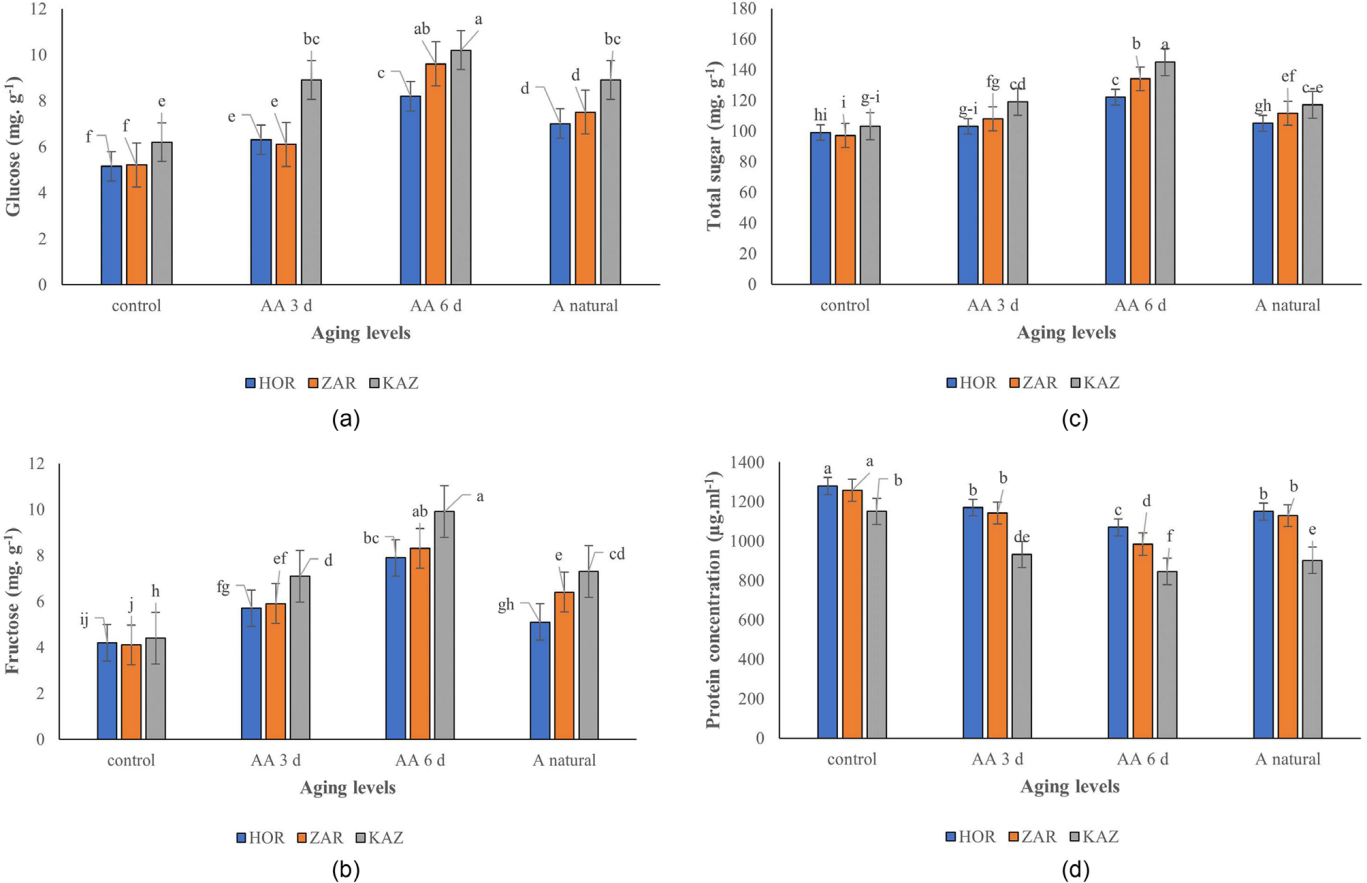
Glucose (**a**), Fructose (**b**), Total sugar (**c**) and Protein concentration (**d**) of Horand, Zargan and Kazerun onion landraces under different levels of aging [Means with the same letters are not significantly different using Duncan multiple range test (*p* value ≤ 0.05)]. AA 3d, AA6 d and A natural refer to three-days aging, six-days aging, and one-year natural aging, respectively

The results indicate that aging, landraces, and their interaction have a significant effect on the seeds’ glucose content. The glucose content increased with increasing aging levels, and this increase was higher in Kazerun than Zargan and Horand landraces. In Kazerun, the glucose content increased by 44%, 65%, and 44% in three days, six days, and one-year natural aging, respectively, compared to the control. In contrast, the glucose content in Horand increased by 22%, 59%, and 36%, compared to the control, respectively. These findings suggest that Horand landrace may be more resilient to aging-induced changes in glucose content compared to the other two seeds (Fig. 3a).

#### Fructose

The levels of fructose in seeds were significantly influenced by aging, landraces, and the interaction between the two (*p* ≤ 0.001). As aging levels increased, the amount of fructose also increased in the seeds, with a more pronounced effect observed in the Kazerun landrace. The fructose content of Horand seeds increased by 21% under normal aging conditions, and by 36% and 88% under accelerated aging conditions of three and six days, compared to the control seeds, respectively. In Zargan landrace, increasing levels of aging resulted in 44%, 102%, and 56% more fructose in seeds under three-day, six-day, and one-year aging conditions, respectively. Conversely, the seeds of Kazerun had 61%, 125%, and 66% higher fructose content compared to the control seeds under the same aging conditions (Fig. 3b).

#### Total soluble sugars

The level of total seeds soluble sugars was significantly influenced by aging, landraces, and their interaction (*p* ≤ 0.001). Total seed soluble sugars content increased with increasing aging levels in all landraces, but Kazerun landrace showed a greater increase than Zargan and Horand (Fig. 3c). The Horand landrace exhibited a respective increase of 4%, 23%, and 6% in total sugar content at three-day, six-day, and one-year natural aging conditions. The Zargan landrace, on the other hand, had an increase of 11%, 38%, and 15% higher than control seeds at the same aging levels. The seeds of Kazerun landrace also showed a significant increase in total sugar content, with respective increases of 16%, 41%, and 15% higher than the control seeds (Fig. 3c). Our study found a positive correlation among glucose, fructose, and total sugar with electrolyte leakage, and a negative correlation with germination percentage and rate, as shown in Fig. 2.

#### Seed protein concentration

The effects of aging, landraces, and their interaction on seed protein concentration were found to be significant (*p* ≤ 0.001). Seed protein concentration decreased at different levels of aging. In Kazerun, the protein concentration decreased by 19%, 26%, and 22%, in three-day aging, six-day aging, and one-year natural aging compared to the control treatment, respectively. Horand seed protein concentration in three-day aging, six-day aging, and one-year natural aging decreased by 9%, 16%, and 10% and by 9%, 22%, and 10% in Zargan, respectively (Fig. 3d). The seed protein concentration was found to have a positive correlation with germination percentage and rate in our study (Fig. 2).

### Antioxidant enzymes activity

**Fig. 4:**
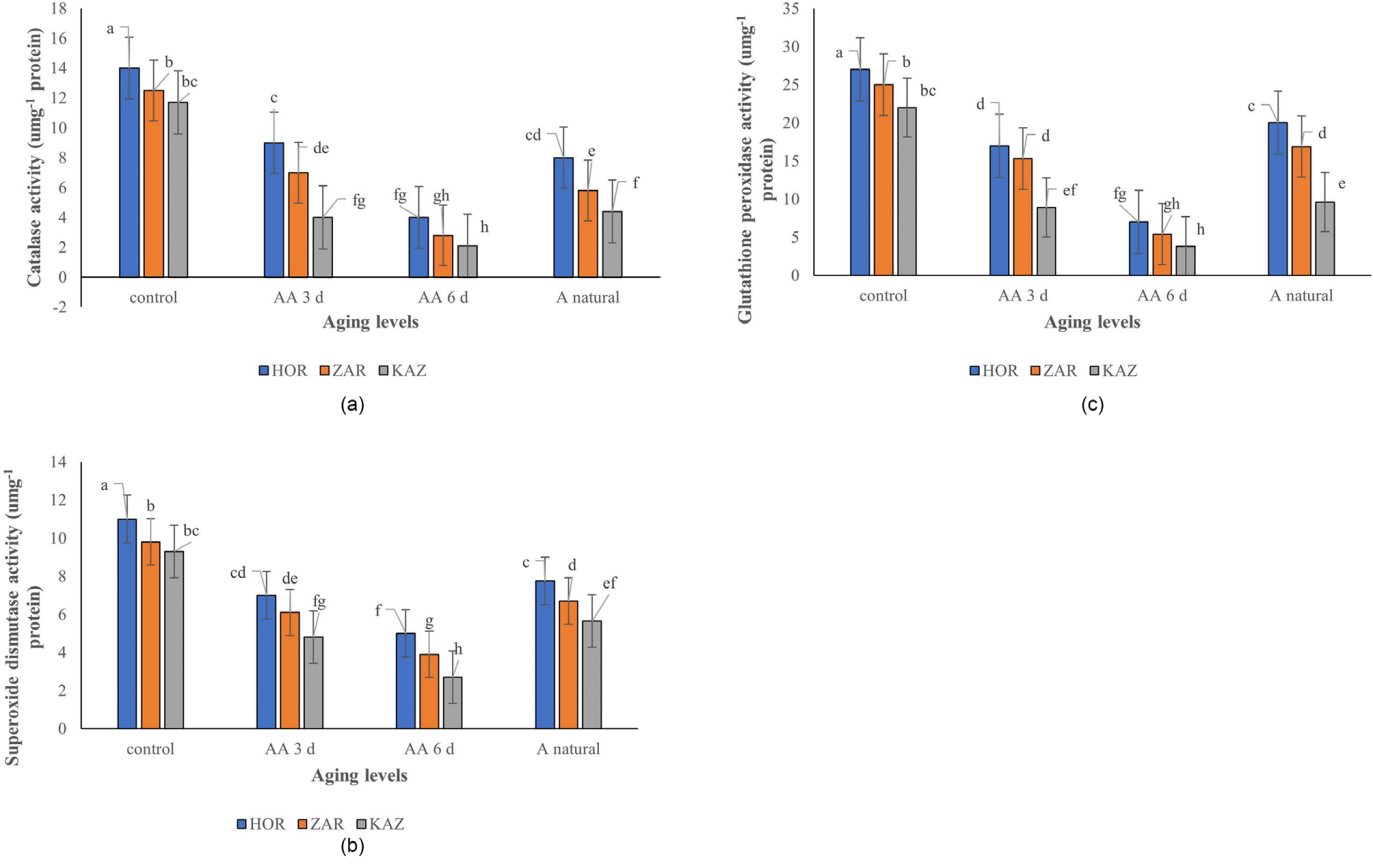
Catalase (**a**), SOD (**b**) and Glutathione peroxidase (**c**) of Horand, Zargan and Kazerun onion landraces under different levels of aging [Means with the same letters are not significantly different using Duncan multiple range test (*p* value ≤ 0.05)]. AA 3d, AA6 d and A natural refer to three-days aging, six-days aging, and one-year natural aging, respectively

The results of the analysis of variance indicate that aging, landrace, and their interaction had a significant effect on superoxide dismutase (SOD) (*p* ≤ 0.001), and glutathione peroxidase activity (*p* ≤ 0.001). Application of different levels of aging reduced the catalase activity of leaves. Horand had higher catalase activity than Zargan and Kazerun at all aging levels. In Kazerun, the activity of catalase decreased by 66%, 82%, and 62% for three-day aging, six-day aging, and one-year natural aging, respectively, compared to the control (Fig. 4a). However, the activity of catalase decreased by 36%, 71%, and 43% for Horand and 44%, 78%, and 54% for Zargan in three-day aging, six-day aging, and one-year natural aging, respectively. Different levels of aging also caused a decrease in SOD activity in onion cultivar and landraces (Fig. 4b). In Kazerun, the SOD activity decreased by 48%, 71%, and 39% after three-day aging, six-day aging, and one-year natural aging, respectively, when compared to the control treatment. Similarly, in Horand, the SOD activity decreased by 36%, 55%, and 30% after three-day aging, six-day aging, and one-year natural aging, respectively, when compared to the control treatment. Glutathione peroxidase also decreased at all aging levels in Horand, Kazerun, and Zargan (Fig. 4c). The glutathione peroxidase activity in the Kazerun decreased by 60%, 83%, and 56% after three-day, six-day, and one-year natural aging, respectively, when compared to the control. However, in Horand, the glutathione peroxidase activity decreased by 37%, 74%, and 26% after three-day aging, six-day aging, and one-year natural aging, respectively, when compared to the control. There was a positive correlation between CAT, SOD, and GPx with the percentage and rate of germination (Fig. 2).

### Gene expression

**Fig. 5:**
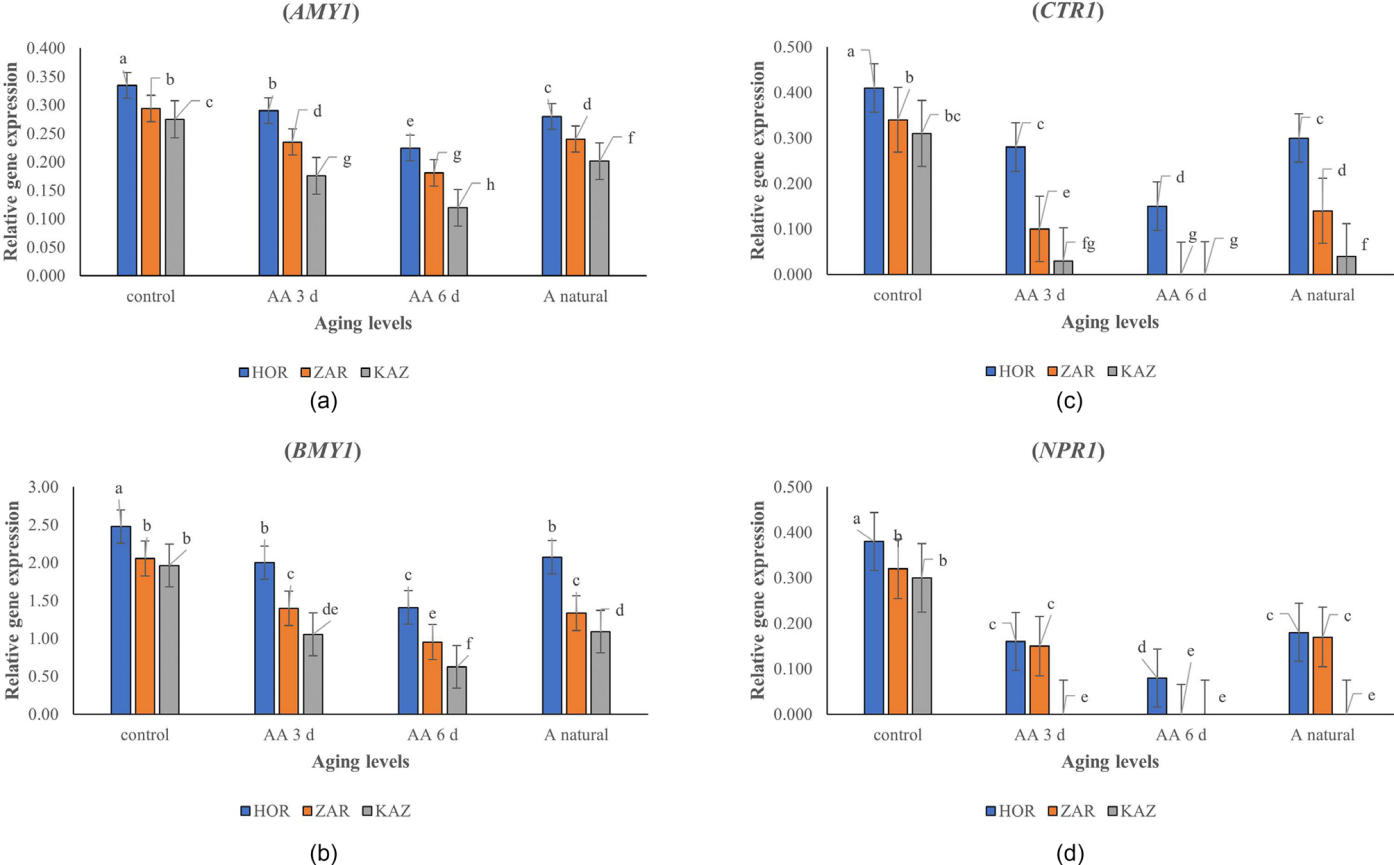
*AMY1* (**a**), *BMYI* (**b**), *CTR1* (**c**) and *NPR1* (**d**) relative gene expression of Horand, Zargan and Kazerun onion landraces under different levels of aging. [Means with the same letters are not significantly different using Duncan multiple range test (*p* value ≤ 0.05)]. AA 3d, AA6 d and A natural refer to three-days aging, six-days aging, and one-year natural aging, respectively

The onion seed aging, landrace, and their interaction imposed a significant effect on the expression of *AMY1*, *BMY1*, *CTR1*, and *NPR1* genes (*p* ≤ 0.001). The results showed that the expression of *AMY1* gene decreased with increasing aging levels in all three landraces, and the highest gene expression was observed in the Horand landrace (Fig. 5a). The expression of *AMY1* decreased in Horand by 13%, 33%, and 16% in three-day, six-day, and one-year natural aging, respectively, and by 20%, 38%, and 18% in Zargan. *AMY1* expression in Kazerun landrace decreased by 36%, 56% and 27%, respectively, in three-day, six-day and one-year natural senescence, compared to the control treatment. *BMY1* expression also decreased with increasing senescence levels in all three landraces (Fig. 5b). In Kazerun, *BMY1* expression decreased by 46%, 68%, and 44% in three-day, six-day, and one-year normal aging, respectively, compared to the control treatment. Similarly, increased aging levels from control to three-day, six-day, and one-year natural aging decreased *BMY1* expression in Horand by 19%, 43%, and 17%. Also, the expression of *BMY* gene in Zargan cultivar in three-day, six-day and one-year natural aging decreased by 32%, 54% and 35% respectively compared to the control. On the other hand, *CTR1* expression decreased with decreasing levels of senescence, although the expression of *CTR1* was almost zero in Kazerun landrace in the six-day aging treatment.

**Fig. 6:**
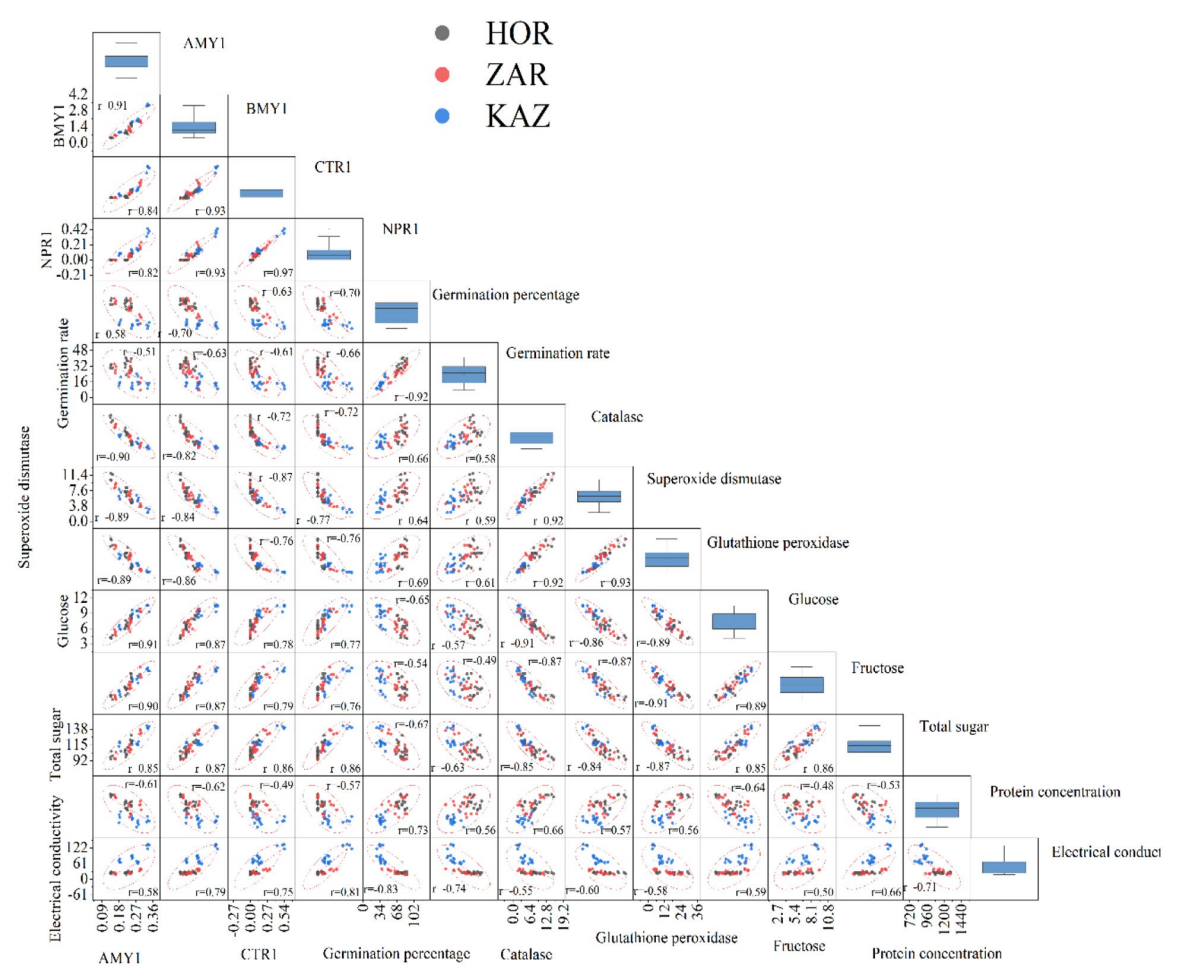
The relationship between germination percentage, germination rate, catalase, superoxide dismutase, glutathione peroxidase, glucose, fructose, total sugar, protein concentration, electrical conductivity, *AMY1*, *BMY1*, *CTR1* and *NPR1* gene expressions of Horand (HOR), Zargan (ZAR) and Kazerun (KAZ) onions landraces under different levels of seed aging

However, gene expression was observed in Horand (Fig. 5c) but in three-day, six-day aging and one-year natural aging, the expression of *CTR1* gene in decreased by 32%, 63% and 27% respectively compared to the control, while in Zargan cultivar, at three-days aging and one year natural aging, *CTR1* gene expression decreased by 71% and 59%, respectively, compared to the control, and this gene was not expressed in six-days aging. The study also found no *NPR1* gene expression in Kazerun in the three-day, six-day, and one-year aging, while, in Horand, three-day, six-day, and one-year normal aging decreased *NPR1* expression by 58%, 79%, and 53%, respectively, compared to the control. On the other hand, the expression of NPR1 gene in Zargan cultivar decreased by 53 and 47% compared to the control at three-day and one-year natural aging, while no gene expression was observed at six-day aging. (Fig. 5d). Additionally, a negative correlation was observed among the expression of these genes and the percentage and rate of germination, as well as with the levels of seed CAT, SOD, GPx, and protein concentration (Fig. 2). This study also revealed a negative correlation between gene expression and levels of seed glucose, fructose, and total soluble sugars (Fig. 6).

## Discussion

Seed decay can lead to various molecular, biochemical, and physiological changes in plants. Depending on their level of resistance, different seeds may respond differently to seed decay. Seeds use various physiological and biochemical strategies, including regenerating cell membrane lipid compounds, to prevent cell damage caused by decay [19].

The results of this study indicate that as aging levels increase, electrolyte leakage also increases, which is associated with a decrease in both germination percentage and rate. However, Kazerun landrace was found to be more sensitive than Horand landrace and Zargan cultivar to aging levels. According to Panayotov and Jadchak [20], low-quality seeds deteriorate much stronger and faster than higher-quality ones. This clearly shows that one of the reasons for reduced germination is the loss in cell membrane integrity [21]. Previous studies have also reported the negative impact of decay on cell membrane integrity [18 and 19]. Studies on wheat (*Triticum aestivum* L.) have also shown that electrolyte leakage increases with aging levels during accelerated aging from one to five days [22]. These results demonstrate that an increase in seed electrolyte leakage caused by membrane decay affected by aging leads to a decrease in the rate and percentage of germination, which is consistent with our results.

Consistent with our findings, existing research shows that the ability of seeds to germinate decreases as catabolic changes occur with age [23]. A decrease in viability or germination capacity does not happen immediately after handling and under optimal storage conditions, the onset of decline in germination may begin several months to several years after seed development, depending on storage conditions [24].

Seed aging is a complex biological trait and involves a network of molecular, biochemical, physiological, and metabolic processes [25]. To avoid misinterpretation that the processes are successive events, it is hypothesized that there is a division of the deterioration into three phases; Phase I is the initial stage after harvest, during which time, deterioration is largely stable [26]. This stage is associated with the depression of the protective capacity against oxidative damages by Amadori and Maillard reactions and minor injuries to the genetic material [19], which do not significantly impact seed viability. In phase II, the deterioration begins with the reduction of the protection capacity against ROS, the lipid peroxidation becomes evident, with damage to the membranes and production of MDA [19]. The ROS and the MDA, in turn, trigger severe damage to the genetic material, generating viable seedlings, but with suppressed growth and an aqueous aspect [19]. In phase III, the viability reduction curve becomes more pronounced. At this stage, the disruption of the mitochondrial membranes increases the respiration due to the lowered production of energy per substrate, since there is a reduction in the efficiency of electron transport [27]. With increased respiration, the ROS production is increased, leading to an autocatalytic cycle with lipid peroxidation, and there is a rise in damage to the genetic material, which ultimately inhibits germination completely [77]. Therefore, the leakage of electrolytes from the membrane is considered as an important index for assessing seed germination [28]. Natural aging in French bean seeds that were stored for more than 4 years was observed to cause the membrane to break and leak UV-absorbing substances, leading to increased electrical conductivity [29].

Our results are consistent with previous studies that have reported soluble sugars increasing during seed aging [11 and 30]. The presence of regenerating soluble sugars, such as glucose, fructose, and galactose, in onion seeds can reduce their potency due to the Amadori and Millard reactions. Orthodox seeds are characterized by the absence of or the presence of a small amount of regenerating soluble sugars [31], which increase with seed age. Studies have also found that the content of glucose and lipid peroxidation products in the axis of *Vigna radiate* seeds increases during storage [32]. The accumulation of Amadori products in seed axils was found to be correlated with lipid peroxidation, while the accumulation of Millard products was closely related to the hydrolysis of soluble sugars [33]. Moreover, soluble sugars, particularly oligosaccharides, and the ratio of oligosaccharides to soluble sugars have been reported to have a positive correlation with seed longevity [34].

The seed protein concentration showed a positive correlation with germination percentage and rate in our study. One possible reason for the decrease in protein content could be the disruption of the protein synthesis system in deteriorated seeds [35]. DNA damage resulting from seed deterioration could also be a contributing factor in the disturbance of protein synthesis [36]. Furthermore, the increase in the activity of catabolic enzymes, such as proteases, could lead to a reduction in protein concentration [35]. In both natural and artificial aging, protein carboxylation increases significantly and the oxidation of proteins is a targeted process, and specific proteins are affected. This process ultimately might leads to a reduction in RNA translation during protein synthesis in both natural and artificial aging [36].

The content and activity of antioxidant enzymes (catalase, glutathione peroxidase, and superoxide dismutase) decreased in all onion landraces under different aging levels. Catalase is an efficient enzyme that breaks down hydrogen peroxide in the embryonic axis [37]. When the onion seed landraces were stored at 40 °C and 100% relative humidity, the seeds experienced stress and lost their germination ability in a time-dependent manner [38]. Our results suggest that catalase and ascorbate peroxidase play an essential role in preventing oxidative stress in onions by catalyzing the reduction of hydrogen peroxide [39]. Based on our results, onion seed germination ability may be related to free radical scavenging efficiency because free radicals may affect storage ability and germination. The effect of accelerated aging on germination ability and physiological characteristics related to peroxidation was investigated in two wheat landraces, and the results showed that accelerated aging inhibits seed germination, seedling growth, and SOD and ascorbate peroxidase activity [40]. Similar to our findings, it was observed that sunflower seeds stored for a long time showed very low glutathione peroxidase activity [41]. Glutathione (in reduced form) and glutathione reductase activity were also low in these seeds, indicating that oxidative damage occurred during aging [41]. Decrease in glutathione peroxidase activity and low germination rate in normal aging onion seeds were associated with hydrogen peroxide accumulation and malondialdehyde content as indicators of peroxidation [42]. It has also been reported that during accelerated senescence of sunflower seeds, lipid peroxidation causes damage to free radical scavenging systems and a decrease in catalase and glutathione reductase activity occurs [34]. Mohaddes Ardebili et al. [43] reported a significant reduction in the content of CAT, APX and SOD activity in severely deteriorated wheat seeds compared to low deteriorated seeds. In another study, about 30% reduction was observed in CAT activity after 7 days of aging compared to the activity of non-aged sunflower seeds [39]. CAT transcripts decreased to an indiscoverable level at 7 days of aging, so it occurred because of the degradation of oxidized RNA after aging-induced ROS storage. Indeed, the content of total extracted RNA from aged seeds was 2.6-fold lower than that of non-aged seeds [39]. Results of another study showed that the aging process of rice seeds did not affect SOD activity levels [44]). SOD does not play a major role in seed aging as already observed by Stewart and Bewley [45] in soybean. Lack of sunflower seed viability at the time of incubation at 45 °C in water or at 100% RH reduced the activities of SOD and CAT. According to our results, a positive link between antioxidant activity and the percentage and rate of germination was observed [46]. El-Maarouf-Bouteau [47] reported that seed senescence disrupted metabolic homeostasis. In aging conditions, plants regulate different metabolic pathways to gain resistance to aging, and antioxidant accumulation is one of the most critical pathways [48]. SOD catalyzes the dismutation of O_2_^−^ to O_2_ and H_2_O_2_ which can then be broken down by other essential enzymes like catalases [49 and 50]. They are recognized as essential defense enzymes against ROS-induced oxidative stress [39]. GRs are extremely specific and are involved in the reduction of oxidized glutathione (GSSG) back to the reduced form (GSH) using NADPH as the reductant [50], thereby sustaining a high GSH to GSSG ratio [51]. They have been demonstrated to enhance oxidative stress tolerance in transgenic *Nicotiana tabacum* [52]. APXs are also involved in the decomposition of H_2_O_2_ using ascorbate as a reductant [53]. GPXs have been demonstrated to play a role in lipid hydroperoxide detoxification, plant defense, and response to biotic [54] and abiotic stresses [55].

The expression of *AMY1*, *BMY1*, *CTR1*, and *NPR1* genes decreased in the response to seed aging and this decrease was more in Kazerun than the other landraces. Previous studies have shown similar decreases in the expression of *AMY1* in rice [56] and *BMY1* in barley [57] under accelerated senescence conditions. Alpha-amylase is synthesized de novo during seed germination in the presence of endogenous GA (gibberellic acid) from the embryo [17]. Conversely, β-amylase is present prior to germination in an inactive form without control of GA [17], which is activated by protease activity that cleaves its carboxyl terminus [58]. In a study, it was detected that one of the alpha-amylase (amylase1A) in the rice seed embryo and demonstrated that the levels of its protein and mRNA both significantly increase and peak at 48 h after imbibition during germination [59]. Consistently, Lin et al. [60] reported that aging remarkably suppressed rice seed germination by increasing GA2ox activity and OsGA2ox5 expression, thereby suppressing GA accumulation in seeds. It was proposed that GA2ox might play a crucial role in the regulation of GA on seed deterioration during seed storage [61]. Therefore, by suppressing the accumulation of GA, the expression of alpha-amylase in the seed is disrupted because these are synthesized in the presence of GA [17]. β-amylase converts starch to UDP-glucose and fructose, which are important for storage function and metabolism [62]. Researchers reported that the starch content in seeds decreased as a result of ageing due to the hydrolysis action of alpha and beta amylase [63]. Studies have shown that CTR1 interacts with ethylene receptors such as ETR1, ETR2 and EIN4 and regulates ethylene intake. CTR1 has been shown to act as a negative regulator of the ethylene signaling pathway [15]. It seems that both ethylene and ROS interplay during germination in stressful conditions [64]. In Arabidopsis, Jurdak et al. [65] showed that the stimulating effect of ethylene on seed dormancy alleviation required ROS production that resulted from the mitochondrial electron transfer chain. Interestingly, they found that ethylene triggered mitochondrial retrograde signaling leading to nuclear ROS production [64]. As a result, the expression of the CTR1 gene, which has a negative effect on ethylene production, decreases in stress conditions (seed decay) with an increase in ethylene. NPR1 is critical in response to stress that is induced by salicylic acid. Salicylic acid causes the monomerization of NPR1 and its transfer from the cytosol to the nucleus. It reacts with other factors in the nucleus and activates the expression of defense genes, thus creating resistance to stress factors [76]. The NPR1 gene expression decreases with the reduction of salicylic acid under aging conditions. This study also revealed that the gene expression and levels of glucose, fructose, and total soluble sugars are mutually influencing, indicating that similar mechanisms of seed decay might be activated in aging conditions across all landraces, but more prominently in Kazerun, leading to greater reductions in germination percentage and rate than in the Zargan and Horand landraces. Additionally, CAT, SOD, GPx enzymes, and protein concentration were positively correlated seed germination percentage and rate, indicating that these metabolites reduce the rate of seed deterioration in all onion landraces.

The molecular processes associated with onion seed aging under different storage conditions have not been fully understood. It is suggested that by studying more genomics, existing ambiguities in this area can be resolved. Furthermore, the role of various priming treatments in seed aging and the regulation of the genes involved could be investigated in the future studies.

## Conclusion

Due to the worldwide significance of onions, it is crucial to screen for onion varieties and landraces that are resistant to seed decay, as well as to understand the mechanisms by which seeds can withstand storage conditions like temperature and humidity. Our study found that storage conditions of onion seeds led to a decrease in CAT, SOD and GPx enzymes, protein concentration and expression of *AMY1*, *BMY1*, *CTR1* and *NPR1* genes, while increasing levels of glucose, fructose, total sugars, and electrolyte leakage. These changes led to a reduction in the percentage and rate of germination. Iranian onion landraces displayed varying responses to different levels of aging, as evidenced by molecular, biochemical, and physiological changes such as antioxidant enzymes, various soluble sugars, protein concentration, and electrolyte leakage. However, different varieties and landraces had different reactions to aging, our findings demonstrate that the Kazerun landrace is more susceptible to storage conditions than the Horand and Zargan landraces, largely due to decreased expression of *AMY1*, *BMY1*, and *CTR1* and *NPR1* genes. This leads to a reduction in the enzyme’s activity of CAT, SOD, and GPx. Moreover, in the Kazerun landrace showed higher levels of glucose, fructose, total sugar and electrolyte leakage than Horand and Zargan landraces. Therefore, Horand and Zargan are more resilient to storage conditions compared to Kazerun.

## Materials and methods

### Plant materials and experimental design

The study was conducted at the plant physiology laboratory of Ferdowsi University of Mashhad, Iran. A 3 × 4 factorial experiment based on completely randomized design with four replications was employed to investigate the effects of different aging levels (control, 3-days accelerated aging, 6-days accelerated aging, and one-year natural aging) on Zargan cultivar, Horand and Kazerun onion landraces. As a released cultivar, Zargan was obtained from Falat Seed Company that has the license of onion seed production, while Horand and Kazerun landraces were kindly offered by the seed bank of the Plant Sciences Research Institute, Ferdowsi University of Mashhad, Iran, with codes MOC101 and MOC102, respectively.

### Seed germination

The standard germination test was conducted according to the rules of the International Seed Testing Association (ISTA) for 12 days, in dark conditions with a temperature of 20 ºC and relative humidity of 60%. The criterion for seed germination was 2 mm length of radicles.

### Seed aging conditions

Seeds were subjected to temperature and humidity stress through accelerated aging, where they were placed in Petri dishes with filter paper and 5 ml of distilled water at a temperature of 40 ºC and relative humidity of 100%.

### Traits measurement

#### Germination characteristics

The total germination percentage was calculated using the following Eq. 1:

1 $$Germination \left(\%\right)=\frac{Ni}{N}\times 100$$

Where Ni is the number of germinated seeds, and N is the number of seeds in the Petri dish.

The germination rate (Gr) was calculated using Maguire’s relation [66] (Eq. 2):

2 $$Gr=\frac{\left(Ni\right)}{\left(days\, to\, first\, count\right)}+\dots +\frac{\left(Ni\right)}{\left(days\, to\, final\, count\right)}$$

#### Electrical conductivity

The electrical conductivity (EC) per gram of seed for each sample was obtained according to the instructions of the ISTA [67] and was calculated using the following Eq. 3:

3 $$EC \left(\mu S{cm}^{-1}{g}^{-1}\right)=\frac{ECs-ECc}{W}$$

W is the weight of the seed sample in grams, ECs is the electrical conductivity of each seed sample in µ-Siemens/cm, ECc is the electrical conductivity of the water in µ-Siemens/cm, and EC is the electrical conductivity obtained in µ-Siemens/cm per gram.

To investigate the physiological traits involved in germination, seeds were used at the physiological germination stage (i.e., the stage before the emergence of the radicle).

#### Measurement of total protein and Soluble sugars

Extraction and measurement of soluble sugars arranged using the phenol-sulfuric acid [78] method with slight modifications. For the extraction of sugars, 0.5 g of powdered seed sample were transferred to 50 mL Falcon tubes. Then, 37.5 mL of 80% ethanol (preheated) was added to the Falcon tubes, followed by one minute of vortexing. Subsequently, the Falcon tubes were centrifuged for 10 min at 3000 rpm at room temperature to separate the liquid phase from the solid phase. The supernatant in the Falcon tubes was transferred to a 200 mL flask. This process was repeated by adding an additional 37.5 mL of preheated 80% ethanol to the remaining residue at the bottom of the Falcon tubes, vortexed, centrifuged, and transferred the supernatant to the 200 mL flask to ensure the extraction of all available sugars. The 200 mL flask was placed in an oven at 45 °C for 48 h for all the ethanol to evaporate.

To measure the total concentration of the extracted sugars, 40 mL of distilled water was added to the flask containing sugar particles, and the flask was shaken to detach all sugar residues from the walls. The sugars easily dissolved in distilled water, but to remove other impurities, 5 mL of 5% zinc sulfate and 4.7 mL of 0.3 N Barium hydroxide were added to the flask and stirred for 4 min on a stirrer. The resulting solution was poured into 50 mL Falcon tubes and centrifuged for 10 min at 3000 rpm to separate the liquid phase from the solid phase. The supernatant from all Falcon tubes related to one sample was transferred to a single flask, and at this stage, they could be stored in a refrigerator at 4 °C. Before reading the concentrations using a spectrophotometer, 2 mL of the solution from each sample was transferred to 15 mL Falcon tubes, and 1 mL of 5% phenol solution was added to each. After closing the Falcon tube caps, they were vigorously shaken until white bubbles formed on the solution’s surface. Then, 5 mL of 98% sulfuric acid was added to the Falcon tubes using a pipette. After allowing the samples to cool off and their colors to stabilize for 45 min to 1 h under a hood, the concentrations of the samples could be measured using a spectrophotometer at a wavelength of 485 nanometers.

Sucrose, glucose, and fructose were examined through HPLC analysis with an RI detector. The HPLC system comprised a binary pump (Perkin Elmer LC-200, Norwalk, CT, USA), an autosampler (Perkin Elmer LC-200), a refractive index-150 detector (System Spectra), and a carbohydrate column known as Rezex^™^ RCM-Monosaccharide Ca^2+^ (8%) measuring 300 × 7.8 mm (Phenomenex, Torrance, CA, USA), which was equipped with a guard cartridge. To inject the sample, a total of 20 µL was utilized. The column temperature was maintained at 80 °C by utilizing a column heater (Jones chromatography, Lakewood, CO, USA). A degassed solvent, water, was employed at a flow rate of 0.6 mL/min, facilitated by a degasser (Gastorr TG-14). The concentrations of sucrose, glucose, and fructose were calculated by employing standard curves within the range of 1.25 to 20 mg/mL.

Equation (4) was used to determine the sugar concentration, where E: sample sugar amount in mg/g dry weight, C: sample sugar concentration in mg/liter, D: degree of dilution, V: final volume of prepared extract, DM: weight of dry matter in grams. 0.5 g).

4 $${\rm{E}}\,{\rm{ = }}\,\left( {{\rm{C}}\,{\rm{ \times }}\,{\rm{D}}\,{\rm{ \times }}\,{\rm{V/}}\,{\rm{DM}}\,{\rm{ \times }}\,{\rm{1000000}}} \right)\,{\rm{ \times }}\,{\rm{1000}}$$

The total protein extraction was performed based on the method described by Bradford [68] with minor modifications. For protein extraction, an extraction buffer was required. To prepare the extraction buffer, 2.423 g of Tris was dissolved in 100 milliliters of distilled water, and the pH of the solution was adjusted to 7.8 using a concentrated hydrochloric acid solution. After 24 h of refrigeration, the pH of the solution was rechecked and adjusted, and 20 milliliters of glycerol were added, bringing the final volume to 200 milliliters. All extraction steps were carried out at a temperature of 4 °C on ice.

A total of 0.25 g of powdered seeds were transferred to a 15-milliliter Falcon tube, and 2.5 milliliters of the extraction buffer were added. The samples were then vortexed and centrifuged at 13,000 rpm for 15 min using a centrifuge at 4 °C. After centrifugation, the supernatant was transferred to another tube, and this obtained extract was used for the determination of total protein, as well as the quantitative measurement of enzyme activities, including catalase (CAT), glutathione peroxidase (GPx), and superoxide dismutase (SOD).

Then the Bradford solution was prepared. To prepare the Bradford solution, 0.1 gram of Coomassie Blue, 50 milliliters of 96% ethanol, and 100 milliliters of 85% orthophosphoric acid were required. Coomassie Blue, ethanol, and a portion of distilled water were poured into an Erlenmeyer flask and stirred using a stirrer. Meanwhile, orthophosphoric acid was gradually added drop by drop. The solution was kept in the refrigerator for several hours. Then, it was brought to a final volume of 1 L using distilled water. The solution was filtered through a filter paper. It should be noted that throughout the preparation of the Bradford solution, it was not exposed to light. In order to construct a standard curve and measure the protein concentrations, 10 milligrams of bovine serum albumin 1 were added to 10 milliliters of distilled water inside a 15-milliliter Falcon tube. By this stage, there was 1 milligram of BSA in each milliliter of this solution. We then prepared concentrations of 0, 300, 600, 900, 1200, and 1500 µg/ml of BSA from the solution. The absorbance of these solutions was measured, and they were used to construct the standard curve. One milliliter of the Bradford solution was poured into 1.5-milliliter tubes, and 40 µl of the seed extract was added to each tube. After 20 min, the absorbance at a wavelength of 595 nanometers was measured using a plate reader.

### Assay of antioxidant enzymes activity

In order to measure the antioxidant enzyme activity, 100 mg of seed sample were homogenized using the ice-cold extraction method with 1000 µl of 0.1 M potassium-phosphate buffer (pH 7.8) containing ethylenediaminetetraacetic acid (EDTA) at one ml concentration. After 20 min of homogenization, the homogenate was centrifuged at 12,000 rpm and a temperature of 4 °C, then the transparent phase was collected [69]. CAT activity was recorded at a temperature of 25 ± 1 °C using the Clairbone’s method [70] and measured at 240 nm. GPx enzyme activity was measured at 25 ± 1 °C and 470 nm according to the method proposed by Polle et al. [71]. SOD activity was measured according to Giannopolitis and Ries’ method [72], and enzyme activity was checked photothermically. The spectrophotometer was calibrated at a wavelength of 560 nm.

### Gene expression

RNA extraction was performed following the method described by Chang et al. [73]. To obtain cDNA, 2 micrograms of RNA was added to a tube containing 1 µl of 5 mM Oligo dT and 2 µl of 5 mM dNTP, and the volume was adjusted to 13 µl. The tubes were incubated at 65 °C for 5 min, then placed on ice for 1 min and centrifuged for 5 s. Next, 4 µl of FSB buffer, 1 µl each of 0.1 mM DTT and 0.1 mM RNase inhibitor, and 1 µl of 1 mM SSIII were added to the tubes. The tubes were then exposed to 50 °C for 60 min, followed by 70 °C for 15 min. After that, 0.5 µl of RNase H was added to the tubes, which were then incubated at 37 °C for 30 min. Finally, MQ H_2_O (Manfactured by DNAbiotech Co. IR Iran) was added to the tubes to reach a final volume of 100 µl, completing the cDNA synthesis.

For qRT-PCR, the cDNA produced in the previous step was diluted 1:10. Then, 10 µl of qRT-PCR Master (Invitrogen Company), 10 µl of diluted cDNA, and 1 µl of primer were used to perform qRT-PCR. The primers were designed using Primer 3 and Oligo analyzer software and are listed in Table 1. In this study, the gene *actin* was selected as an internal control [74]. A Gene Q rotor machine (Qiagen) was used to perform the qRT-PCR.

**Table 1:** Primers forwards and Reverse

	Sequence (5'–>3')	
Gene	Forward primer	Reverse primer
*αMY1*	CCCTGCTCGTACTTGTGTGG	GGGAGAGGTTGTGGGTTTGA
*βMY1*	TGGAGGGAACGTAGGAGATATAG	CTGAGCGGTTGGTGTAGAAG
*CTR1*	ATTCAACCATTCCCCCTGATACT	TTCCATCATCGCAGTGTGTTC
*NPR1*	ACGCTTCTTCCCTCGATGCT	CTCCACATACCTTCTTCGCTTCC
*actin*	CGATGAAGCACAATCCAAGA	TGTTCTTCAGGAGCAACACG

The qRT-PCR procedure comprised of several steps. First, the samples were heated at 94 °C for 4 min. Then, 50 cycles were performed, consisting of 10 s at 95 °C, 15 s at 60 °C, and 20 s at 72 °C. After each cycle, a melting curve program was carried out, starting from 72 °C and increasing to 95 °C with a 5-second delay at each temperature. Fluorescence results were recorded at the 72 °C stage during the melting curve program.

The expression levels of the target genes were compared to the expression level of the reference gene using comparative quantitative analysis with the Rotor-Gene-Q Series software. To control the results and ensure that the initiator dimer formation did not influence the outcomes, a control treatment was used in which water was used instead of the sample.

The data obtained from real-time polymerase chain reaction (PCR) reactions (Ct values) were stored in Excel, and the relative expression of the target genes compared to the reference gene was calculated using Microsoft Excel using the Livak formula. The Livak formula is equivalent to 2^-ΔΔCt, where ΔΔCt is defined as: ΔCt (target gene) - ΔCt (calibrator) [75].

Statistical analysis was conducted using ANOVA with Proc GLM in SAS version 9.4, and mean comparisons were applied using Duncan’s multiple range test with a significance level of *p* ≤ 0.05. Correlation analysis was performed using the corrplot package in R version 4.0.2.

## Data Availability

Data can be provided upon reasonable request from the corresponding author.

## Abbreviations

AMY1: Alpha-amylase 1
BMY1: Beta-amylase 1
CTR1: Constitutive triple response 1
NPR1: Non expressor of pathogenesis related genes
CAT: Catalase
SOD: Superoxide dismutases
GPx: Glutathione peroxidase
ANOVA: Analysis of variance
GLM: Generalized Linear Models
BSA: Bovine Serum Albumin

## Footnotes

1 Bovine Serum Albumin (BSA).

## References

[1] Rosinska A (2022). Evaluation of the use of oregano and coconut hydrolates to improve onion seed quality. Agron.

[2] Abdelkader M, Voronina L, Puchkov M, Shcherbakova N, Pakina E, Zargar M, Lyashko M (2023). Seed priming with exogenous amino acids improves germination rates and enhances photosynthetic pigments of onion seedlings (*Allium cepa* L). Horticulturae.

[3] Khokhar K. Part 1 Chap. 2 Onion: Seed viability and germination. In onion—An ancient crop and modern practices; Noor publishing: Pakistan, 2019.

[4] Tan JW, Kester ST, Su K, Hildebrand DF, Geneve RL (2022). Seed priming and pericarp removal improve germination in low-germinating seed lots of industrial hemp. Crops.

[5] Zhang K, Zhang Y, Sun J, Meng J, Tao J (2021). Deterioration of orthodox seeds during ageing: influencing factors, physiological alterations and the role of reactive oxygen species. int j mol sci.

[6] Collin F (2019). Chemical basis of reactive oxygen species reactivity and involvement in neurodegenerative diseases. Int J Mol Sci.

[7] Adetunji AE, Adetunji TL, Varghese B, Sershen Pammenter NW (2021). Oxidative stress, ageing and methods of seed invigoration: an overview and perspectives. Agron.

[8] Tang J, Wang SQ, Hu KD, Huang ZQ, Li YH, Han Z, Chen XY, Hu LY, Yao GF, Zhang H (2019). Antioxidative capacity is highly associated with the storage property of tuberous roots in different sweet potato cultivars. Sci Rep.

[9] Khan MN, Li Y, Khan Z, Chen L, Liu J, Hu J, Wu H, Li Z (2021). Nanoceria seed priming enhanced salt tolerance in rapeseed through modulating ROS homeostasis and α-amylase activities. J Nanobiotechnol.

[10] Brar NS, Kaushik P, Dudi BS (2019). Assessment of natural ageing related physio-biochemical changes in onion seed. Agriculture.

[11] Nigam M, Mishra AP, Salehi B, Kumar M, Sahrifi-Rad M, Coviello E, Iriti M, Sharifi-Rad J (2019). Accelerated ageing induces physiological and biochemical changes in tomato seeds involving MAPK pathways. Sci Hort.

[12] Hajiabbasi M, Tavakkol Afshari R, Abbasi A, Kamaei R (2020). The effect of ACC and salicylic acid on germination and GAI1 and LOX2 genes expression in deteriorated soybean seeds (*Glycine max*). Iran J Seed Res.

[13] Nadarajan J, Walters C, Pritchard HW, Ballesteros D, Colville L (2023). Seed longevity—the evolution of knowledge and a conceptual framework. Plants.

[14] Hajiabbasi M, Tavakkol afshari R, Abbasi A, Kamaei R (2021). Effects of salicylic acid and ethylene on the germination and gene expression of alpha and beta amylase in deteriorated soybean seeds (*Glycine max*). Iran J Seed Res Tech.

[15] Corbineau F, Xia Q, Bailly C, El-Maarouf-Bouteau H (2014). Ethylene, a key factor in the regulation of seed dormancy. Front Plant Sci.

[16] McGivern JJ, Ganger MT, Ewing SJ (2013). FT and NPR1 expression patterns in Arabidopsis thaliana during flowering and in response to salicylate. Preliminary Rep Bios.

[17] Damaris RN, Lin Z, Yang P, He D (2019). The rice alpha-amylase, conserved regulator of seed maturation and germination. Int J Mol Sci.

[18] Chaudhari HA, Mahatma MK, Antala V (2023). Ethrel-induced release of fresh seed dormancy causes remodelling of amylase activity, proteomics, phytohormone and fatty acid profile of groundnut (*Arachis hypogaea* L). Physiol Mol Biol Plants.

[19] Ebone LA, Caverzan A, Chavarria G (2019). Physiologic alterations in orthodox seeds due to deterioration processes. Plant Physiol Biochem.

[20] Panayotov N, Jadchak D (2020). Genotypic response of different pepper varieties to the accelerated aging test of the seeds. Sci Pap-Ser B-Hortic.

[21] Yan H, Jia S, Mao P (2020). Melatonin priming alleviates aging-induced germination inhibition by regulating β-oxidation, protein translation, and antioxidant metabolism in oat (*Avena sativa* L.) Seeds. Int J Mol Sci.

[22] Shaaban M (2016). The effect of ageing on antioxidant and biochemical changes in wheat (*Triticum aestivum* L.) seeds. Iran J Plant Physiol.

[23] Rao D, Pashwan VR, Chaitanya G, Verma B, Negi PS, et al. Physiological and biochemical changes during seed deterioration in seed –A Review. Deepak Rao., ELSJ. 2023;9(1):1–10. 10.5281/zenodo.7539663.

[24] Wawrzyniak M, Michalak M, Chmielarz P (2020). Effect of different conditions of storage on seed viability and seedling growth of six European wild fruit woody plants. Ann for Sci.

[25] Fu Y, Ahmed Z, Diederichsen A. Towards a better monitoring of seed ageing under ex situ seed conservation. Conserv. Physiol. 2015;1;3(1):cov026 10.1093/conphys/cov026. 10.1093/conphys/cov026 PMC4778438 27293711

[26] Marcos-Filho J (2015). Seed vigor testing: an overview of the past, present and future perspective. Sci Agric.

[27] Xin X, Tian Q, Yin G, Chen X, Zhang J, Nq S (2014). Reduced mitochondrial and ascorbate–glutathione activity after artificial ageing in soybean seed. J Plant Physiol.

[28] Panobianco M, Vieira RP (2007). Electrical conductivity and deterioration of soybean seeds exposed to different storage conditions. Revista Brasileira De Sementes.

[29] Pandey KK (1988). Priming induced repair in French bean seeds. Seed Sci Technol.

[30] Gianella M, Doria E, Dondi D, Milanese C, Gallotti L, Börner A, Zannino L, Macovei A, Pagano A, Guzzon F, Biggiogera M, Balestrazzi A (2021). Physiological and molecular aspects of seed longevity: exploring intra-species variation in eight *Pisum sativum* L. accessions. Physiol Plant.

[31] Berjak P, Pammenter NW (2013). Implications of the lack of desiccation tolerance in recalcitrant seeds. Front Plant Sci.

[32] López-Fernández MP, Moyano L, Correa MD, Vasile F, Burrieza HP, Maldonado S (2018). Deterioration of willow seeds during storage. Sci Rep.

[33] Murthy UM, Sun WQ (2000). Protein modification by amadori and maillard reactions during seed storage: roles of sugar hydrolysis and lipid peroxidation. J Exp Bot.

[34] Corbineau F (2012). Markers of seed quality: from present to future. Seed Sci Res.

[35] Coolbear P. Mechanisms of seed deterioration, Seed quality: basic mechanisms and agricultural implications. 1995;22:223–277.

[36] Job C, Rajjou L, Lovigny Y, Belghazi M, Job D (2005). Patterns of protein oxidation in Arabidopsis seeds and during germination. Plant Physiol.

[37] Wojtyla Ł, Lechowska K, Kubala S, Garnczarska M (2016). Different modes of hydrogen peroxide action during seed germination. Front Plant Sci.

[38] Smith TM (2006). Seed priming and smoke water effects on germination and seed vigor of selected low-vigor forage legumes. Master of science in crop and soil environmental sciences.

[39] Kibinza S, Bazin J, Bailly C, Farrant JM, Corbineau F, El-Maarouf-Bouteau H (2011). Catalase is a key enzyme in seed recovery from ageing during priming. Plant Sci.

[40] Zhang L, Gao B (2021). Effect of Isosteviol on wheat seed germination and seedling growth under cadmium stress. Plants (Basel).

[41] Morscher F, Kranner I, Arc E, Bailly C, Roach T (2015). Glutathione redox state, tocochromanols, fatty acids, antioxidant enzymes and protein carbonylation in sunflower seed embryos associated with after-ripening and ageing. Ann Bot.

[42] Dong L, Hao Z, Li Z, Zhu J, Wang Q (2014). Enhancement of Welsh onion (*Allium fistulosum* L.) seed vigor by KNO3 priming. J Agric Sci Technol.

[43] Mohaddes Ardebili Z, Abbaspour H, Tavakkol Afshari R, Nabavi Kalat SM (2019). Evaluation of germination and antioxidant activity in GA3-primed deteriorated wheat seed. Russ J Plant Physiol.

[44] Yin G, Xin X, Song C, Chen X, Zhang J, Wu S, Li R, Liu X, Lu X (2014). Activity levels and expression of antioxidant enzymes in the ascorbate–glutathione cycle in artificially aged rice seed. Plant Physiol Biochem.

[45] Stewart RC, Bewley JD (1980). Lipid peroxidation associated with accelerated aging of soybean axes. Plant Physiol.

[46] Tan BL, Norhaizan ME, Liew WPP, Sulaiman Rahman H (2018). Antioxidant and oxidative stress: a mutual interplay in age-related diseases. Front Pharmacol.

[47] El-Maarouf-Bouteau H (2022). The seed and the metabolism regulation. Biology.

[48] Kurek K, Plitta-Michalak B, Ratajczak E (2019). Reactive oxygen species as potential drivers of the seed aging process. Plants.

[49] Govindaraj M, Masilamani P, Albert VA, Bhaskaran M (2017). Role of antioxidant in seed quality—A review. Agric Rev.

[50] Perl-Treves R, Perl A. Oxidative stress: An introduction. In oxidative stress in plants; Inzé, D., Van Montagu, M., Eds.; Taylor & Francis: London, UK, 2002; pp. 1–32.

[51] Yousuf PY, Hakeem KUR, Chandna R, Ahmad P, Ahmad P, Prasad MNV (2012). Role of glutathione reductase in plant abiotic stress. Abiotic stress responses in plants.

[52] Yoshimura K, Miyao K, Gaber A, Takeda T, Kanaboshi H, Miyasaka H, Shigeoka S (2004). Enhancement of stress tolerance in transgenic tobacco plants overexpressing Chlamydomonas glutathione peroxidase in chloroplasts or cytosol. Plant J.

[53] Ozyigit II, Filiz E, Vatansever R, Kurtoglu KY, Koc I, Öztürk MX, Anjum NA (2016). Identification and comparative analysis of H2O2-scavenging enzymes (ascorbate peroxidase and glutathione peroxidase) in selected plants employing bioinformatics approaches. Front Plant Sci.

[54] Navrot N, Collin V, Gualberto J, Gelhaye E, Hirasawa M, Rey P, Knaff DB, Issakidis E, Jacquot JP, Rouhier N (2006). Plant glutathione peroxidases are functional peroxiredoxins distributed in several subcellular compartments and regulated during biotic and abiotic stresses. Plant Physiol.

[55] Bela K, Horváth E, Gallé Á, Szabados L, Tari I, Csiszár J (2015). Plant glutathione peroxidases: emerging role of the antioxidant enzymes in plant development and stress responses. J Plant Physiol.

[56] Ouyang N, Sun X, Tan Y, Sun Z, Yu D, Liu H, Liu C, Liu L, Jin L, Zhao B, Yuan D, Duan M (2021). Senescence-specific expression of RAmy1A accelerates non-structural carbohydrate remobilization and grain filling in rice (*Oryza sativa* L.). Front. Plant Sci.

[57] Puchta M, Groszyk J, Małecka M, Koter MD, Niedzielski M, Rakoczy-Trojanowska M, Boczkowska M (2021). Barley seeds miRNome stability during long-term storage and aging. Int J Mol Sci.

[58] Buchanan BB, Gruissem W, Jones RL. Biochemistry and molecular biology of plants; American Society of Plant Physiologists: Rockville, MD, USA. 2015.

[59] He D, Han C, Yao J, Shen S, Yang P (2011). Constructing the metabolic and regulatory pathways in germinating rice seeds through proteomic approach. Proteomics.

[60] Lin C, Shen HQ, Guan YJ, An JY, Hu WM, Hu J (2017). Changes of physiological, biochemistry and gene expression related to ABA and GA3 in hybrid rice seeds stored at different moisture contents and packing methods. Plant Physiol J.

[61] Huang Y, Lu M, Wu H, Zhao T, Wu P, Cao D (2021). High drying temperature accelerates sunflower seed deterioration by regulating the fatty acid metabolism, glycometabolism, and abscisic acid/gibberellin balance. Front Plant Sci.

[62] Song X, Yang Z, Zhang D, Zhang X, Zhang F, Liu J, Yu C (2023). Proteomic analysis of the effect of accelerated ageing on allium mongolicum seeds. Horticulturae.

[63] Lakshmi CJ, Jijeesh CM, Seethalakshmi KK (2021). Impact of accelerated aging process on seed quality and biochemical changes of dendrocalamus sikkimensis gamble. Acta Physiol Plant.

[64] Bailly C, Jurdak R, Corbineau F. Ethylene in the regulation of seed dormancy and germination: molecular mechanisms. The Plant Hormone Ethylene. 2023;41–60. 10.1016/B978-0-323-85846-5.00016-3.

[65] Jurdak R, Launay-Avon A, Paysant-le Roux C, Bailly C (2021). Retrograde signaling from the mitochondria to the nucleus translates the positive effect of ethylene on dormancy breaking of Arabidopsis thaliana seeds. New Phytol.

[66] Maguire JD (1962). Rate of germination-aid selection and evaluation for seedling emergence and vigor. Crop Sci.

[67] International Seed Testing Association. International rules for seedtesting. 2020;i–2–44(44). 10.15258/istarules.2020.02.

[68] Bradford MM (1976). A dye binding assay for protein. Ann Clin Biochem.

[69] Sairam RK, Deshmukh PS, Saxena DC (1998). Role of antioxidant systems in wheat genotype tolerance to water stress. Biol Plant.

[70] Clairbone A. Cataiase activity -. Handbook of methods for oxygen radical research. Ed, Greenwald, R.A (Boca Raton. CRC Press; 1985. pp. 283–4.

[71] Polle A, Otter T, Seifert F (1994). Apoplastic peroxidases and lignification in needles of Norway spruce (*Picea abies* L). Plant Physiol.

[72] Giannopolitis CN, Ries SK (1977). Superoxide dismutases, I, occurrence in higher plants. Plant Physiol.

[73] Chang S, Puryear J, Cairney K (1993). A simple and efficient method for isolating RNA from pine trees. Plant Mol Biol Rep.

[74] Jamshidi Goharrizi K, Dayton Wilde H, Amirmahani F, Mehdi Moemeni M, Zaboli M, Nazari M, Saeed Moosavi S, Jamalvandi M (2018). Selection and validation of reference genes for normalization of qRT-PCR gene expression in wheat (*Triticum durum* L.) under drought and salt stresses. J Genet.

[75] Livak KJ, Schmittgen TD (2001). Analysis of relative gene expression data using real-time quantitative PCR and the 2- ∆∆Ct. Method.

[76] Rivas-San Vicente M, Javier Plasencia J (2011). Salicylic acid beyond defence: its role in plant growth and development. J Exp Bot.

[77] Bin Arif A, Yuliani S, Hernani Q, Agustinisar I, Winarti C. Effects of Chitosan Nanoparticles Coating on Delaying of Seed Soybean (*Gycine Max*) Deterioration. 2023. Emir J Food Agric. 2023;35:3. 10.9755/ejfa. 2023.v35.i3.2982.

[78] Chow PS, Landhäusser SM. A method for routine measurements of total sugar and starch content in woody plant tissues. Tree Physiol. 2004;24(10):1129–36. 10.1093/treephys/24.10.1129. PMID: 15294759. 10.1093/treephys/24.10.1129 15294759

